# Germline variants in MRE11/RAD50/NBN complex genes in childhood leukemia

**DOI:** 10.1186/1471-2407-13-457

**Published:** 2013-10-05

**Authors:** Maria Mosor, Iwona Ziółkowska-Suchanek, Karina Nowicka, Agnieszka Dzikiewicz-Krawczyk, Danuta Januszkiewicz–Lewandowska, Jerzy Nowak

**Affiliations:** 1 Department of Molecular Pathology, Institute of Human Genetics Polish Academy of Sciences, Strzeszyńska 32, 60-479, Poznań, Poland; 2 Pediatric Oncology, Hematology and Bone Marrow Transplantation Department, Poznań University of Medical Sciences, Poznań, Poland

## Abstract

**Background:**

The *MRE11*, *RAD50*, and *NBN* genes encode proteins of the MRE11-RAD50-NBN (MRN) complex involved in cellular response to DNA damage and the maintenance of genome stability. In our previous study we showed that the germline p.I171V mutation in *NBN* may be considered as a risk factor in the development of childhood acute lymphoblastic leukemia (ALL) and some specific haplotypes of that gene may be associated with childhood leukemia. These findings raise important questions about the role of mutations in others genes of the MRN complex in childhood leukemia. The aim of this study was to answer the question whether *MRE11* and *RAD50* alterations may be associated with childhood ALL or AML.

**Methods:**

We estimated the frequency of constitutional mutations and polymorphisms in selected regions of *MRE11*, *RAD50*, and *NBN* in the group of 220 children diagnosed with childhood leukemias and controls (n=504/2200). The analysis was performed by specific amplification of region of interest by PCR and followed by multi-temperature single-strand conformation polymorphism (PCR-MSSCP) technique. We performed two molecular tests to examine any potential function of the detected the c.551+19G>A SNP in *RAD50* gene. To our knowledge, this is the first analysis of the *MRE11, RAD50* and *NBN* genes in childhood leukemia.

**Results:**

The frequency of either the AA genotype or A allele of *RAD50*_rs17166050 were significantly different in controls compared to leukemia group (ALL+AML) (*p*<0.0019 and *p*<0.0019, respectively). The cDNA analysis of AA or GA genotypes carriers has not revealed evidence of splicing abnormality of *RAD50* pre-mRNA. We measured the allelic-specific expression of G and A alleles at c.551+19G>A and the statistically significant overexpression of the G allele has been observed. Additionally we confirmed the higher incidence of the p.I171V mutation in the leukemia group (7/220) than among controls (12/2400) (*p*<0.0001).

**Conclusion:**

The formerly reported sequence variants in the *RAD50* and *MRE11* gene may not constitute a risk factor of childhood ALL in Polish population. The *RAD50*_rs17166050 variant allele is linked to decreased ALL risk (*p*<0.0009, OR=0.6358 (95%CI: 0.4854-0.8327)). Despite the fact that there is no splicing abnormality in carriers of the variant allele but an excess of the G over the A allele was consistently observed. This data demonstrate that some specific alternations of the *RAD50* gene may be associated with childhood ALL.

## Background

Leukemia has the highest incidence rate among the childhood neoplasms [1]. A variety of factors are etiologically involved in leukemia, not only in different cases but also within individual cases. The contribution of inherited genetic susceptibility to the development of cancer is increasingly investigated.

The *MRE11*, *RAD50*, and *NBN* genes encode proteins of the MRN complex involved in the repair of DNA double-strand breaks and other critical cellular functions including telomere maintenance, immunoglobulin class switching, meiotic recombination and DNA damage checkpoint activation [2]. Hypomorphic, biallelic mutations in the *MRE11*, *RAD50* and *NBN* genes are linked to recessive genetic conditions, ataxia telangiectasia like disorder (AT-LD), NBS-like and Nijmegen breakage Syndrome (NBS), respectively, [3-5] some of which are characterized by increased risk of cancer. On the basis of the central role of the MRN complex in the maintenance of genomic integrity, germline heterozygous mutations affecting genes of the MRN complex might play a role in carcinogenesis. In our previous study we showed that the germline p.I171V mutation in *NBN,* one of the MRN genes, may be considered as a risk factor in the development of childhood acute lymphoblastic leukemia [6] and solid malignant tumors including breast cancer, larynx and colorectal cancer in adult [7-9]. Likewise, heterozygous carriers of the *NBN* c.657del5 mutation present increased risk of malignant tumor development, especially breast cancer [10], prostate [11], and colorectal cancer [10]. The *NBN* polymorphisms, especially p.E185Q, have been investigated in some cancer but results were inconclusive [12]. On the other hand some specific *NBN* haplotypes have been related to increased susceptibility to childhood acute leukemia [13]. These findings raise important questions about the role of mutations and polymorphisms in other genes of the MRN complex in the most common malignancies in children. To date, the association of the molecular variants in the *RAD50* and *MRE11* gene with the cancer risk has not been so extensively studied. Although, the germline c.687delT mutation in *RAD50* has been linked to sporadic breast cancer in the Finnish population [14,15], our results excluded the mutation as a risk variant in Polish breast cancer patients [16]. Other molecular variants in the *RAD50* gene: p.I94L in exon 3 and p.R224H in exon 5, intronic variant IVS3-1G>A and a nonsense mutation p.Q350X in exon 7 have been observed at a low frequency not allowing to determine whether the variants increased the risk of breast cancer. Several missense mutations and molecular variants of the *MRE11* gene have been identified in various tumors. Three missense mutations p.S104C, p.R503H and p.R572Q, located at conserved positions of the protein, have been found in breast and lymphoid tumors. Additionally, an aberrant transcript without genomic mutation has been found in a breast tumor [17]. Another group of researchers discovered the p.R305W mutation in the group of 151 patients from the families where a relative had breast cancer and/or ovarian cancer [18]. In the same exon, the p.H302Y variant has been reported in breast cancer [19]. To our knowledge, there is no evidence on the association between the *MRE11* and *RAD50* gene mutation and childhood acute leukemia. With this in mind, we decided to simultaneously analyze the alterations in *MRE11*, *RAD50* and *NBN* genes in Polish children with acute leukemia.

## Methods

### Materials

Two hundred twenty blood samples were collected from children (aged 1–18 years), who were diagnosed with leukemia and were hospitalized at the Pediatric Oncology, Hematology and Bone Marrow Transplantation Department in Poznań. The diagnosis of leukemia was made according to the French-American-British criteria, after conventional cytochemical and surface-marker analysis. 188 children were diagnosed with acute lymphoblastic leukemia (ALL). Approximately 92% of the cases were of the B-cell precursor type, of these, 137 patients expressed the CD10 antigen, 25 had a pre-B immunophenotype, 12 patients had B-cell leukemia and 14 patients, T-cell leukemia. 32 children were diagnosed with acute myelogenous leukemia (AML). Using the FAB criterion, 15 of the patients with AML were of M1 morphology, 9 had M2, 3 had M2/M3, and 5 had M0, M2/M3, M4 and M5, respectively.

The leukemia group was pooled from patients from our previous studies [6,13] and the present work. About 80% of the venous blood samples were obtained from the patients in remission. Anonymous blood samples collected on Guthrie cards drawn from the newborn screening program were used as controls. The leukemia and control samples were recruited from the ethnically homogenous population living in the Wielkopolska province in Poland. The DNA isolation procedure was described in our previous study [6]. To confirm the germline origin of the detected variant we analyzed the DNA from oral epithelium cells.

From all patients participating in the study or from their parents in case of minors, informed consent for participation to permit the scientific using their samples was obtained. The use of the anonymous Guthrie cards as control and the study protocol were approved by the ethics committee at the Poznan University of Medical Sciences.

### Methods

Molecular variants analysis in the MRN complex genes was performed on DNA samples isolated from 220 children diagnosed with leukemia and 504 controls. All samples were analyzed by PCR multi-temperature single-strand conformation polymorphism (PCR-MSSCP) technique (Biovectis, Warsaw, Poland). The exons 2, 5, 6, 7, 10, 13 of the *NBN* gene and the exons 3, 4, 5 and 7 of the *RAD50* gene were analyzed as described in our previous studies [6,16]. Screening of the regions of the *MRE11* was performed using the primers flanking the exons 5, 9, 10, 14, 15, 19 and exon/intron boundaries (primer sequences as Additional file 1). The selection of the screened regions was based on the reported occurrence of the mutations in cancer in former studies. Polymerase chain reaction products were mixed with loading buffer, denatured, cooled and separated on non-denaturing polyacrylamide gel in DNA POINTER PLUS System (Biovectis, Warsaw, Poland) depending on PCR products or digests size. Control samples were run in parallel. Silver staining was used for detection of single-strand DNA in polyacrylamide gels. Each sample showing shifts in multi-temperature single strand conformation polymorphism analyses was subsequently sequenced (OLIGO, IBB, Warszawa).

The RNA of the carriers of each genotype of the c.551+ 19 G>A variant in *RAD50* gene was isolated from blood samples (QIAamp RNA Blood Mini Kit, Qiagen, Germany) according to the manufacturer's protocol. Genotypes were determined by a Taqman genotyping assay (C_33291484_10) (Applied Biosytsem, Foster City, US). RNA was reverse transcribed using the QuantiTect Reverse Transription Kit (Qiagen, Hamburg, Germany). The resulting cDNA pools were used for amplification of the *RAD50* gene transcripts. In order to detect transcripts which may splice out exon 4 or 5 we applied the forward primer 5′AGCCCAGATTCGTCTGCAAT and reverse primer 5′TCTTTCGGCTATCCAAGGCT located in exons flanking exons 4 and 5 and yielding the 602 bp PCR product*.* Amplification reactions were performed in a volume of 25 μl containing AmpliTaq® DNA Polymerase, buffer with 25 mM MgCl_2_ (Applied Biosytem, Foster City, USA), deoxyribonucleotide triphosphates (Sigma-Aldrich, Steinheim, Germany), primers (Oligo, Warszawa, Poland) and cDNA or genomic DNA.

Allele-specific real time PCR was performed to compare expression level from the reference allele and mutant allele of the c.551+19G>A variant (differential allelic expression, DAE). The cDNA and genomic DNA samples (n=30) from heterozygous carriers of the c.551+19G>A variant were amplified with the same abovementioned Taqman assay. The allele G and A were labeled by a different dye and fluorescence was detected on CFX96 Touch™ Real-Time PCR Detection System (Bio-Rad, California, USA). As standard we used a heterozygous genomic DNA sample serially diluted of 100, 20, 4, 0.8, 0.16 and 0.03 ng per reaction. For each sample the real time PCR was performed in quadruple. The ratio G_quantity_/A_quantity_ was used to express differential allelic expression. The robustness and sensitivity of this approach have been shown by many researchers [20-22].

We used the tools available on Ensembl database (ENCODE: software Genomic Context and Variant Effect Predictor) in order to carry out an *in silico* analysis of the detected variants in the *RAD50* gene [23-25].

Statistical analysis was performed with Fisher’s exact test (two-tailed) or chi-square test to evaluate the statistical differences in variants and polymorphisms between the studied group and controls, depending on variant frequency. Crude odds ratios (ORs) were calculated and given with 95% confidence intervals (CIs). The differences were considered significant if the value of probability (*p*) was less than 0.05. In case of polymorphisms, the wild-type genotype/allele served as a reference category. Genotype frequencies observed and expected from the Hardy-Weinberg equilibrium were compared with the standard chi-square test. The results from allele specific real time PCR were analyzed using the Wilcoxon signed-ranks test (http://www.vassarstats.net/wilcoxon.html) to investigate whether the DAE deviated from the null hypothesis.

## Results

In the selected fragments of the *RAD50* and *MRE11* genes, which have been reported to harbor mutations in cancer, we were unable to confirm the occurrence of the formerly known mutations in our group of either ALL or AML cases.

**Figure 1:**
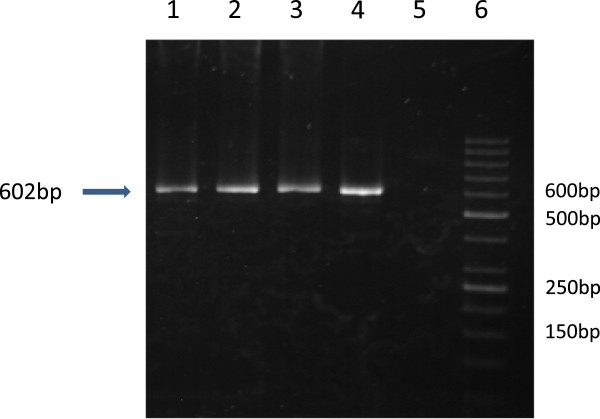
**Analysis of *RAD50 *c.551+19G>A polymorphisms.** Amplification results from four different cDNA samples. Lane 1- the wild type cDNA template. Lane 2- cDNA from the carrier of the c.551+19G>A variant. Lane 3- cDNA from the carrier of the c.551+19G>A variant. Lane 4-cDNA from the carrier of the AA genotype of the *RAD50*_c.551+19. Lane 5-negative control. Lane 6-DNA marker (Gene Ruler 50 bp, *Fermentas*).

**Figure 2:**
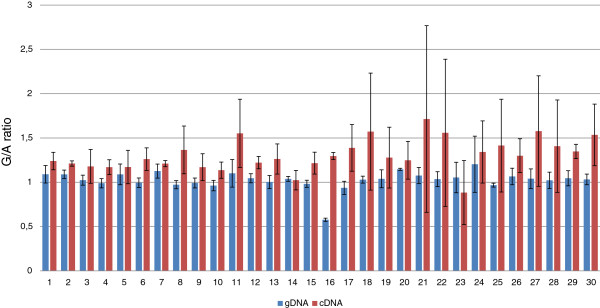
**The ratio of G/A alleles.** Two bars represents one individual (heterozygous carrier of the c.551+ 19G>A), the red one represent ratio of G/A in cDNA, the blue one in gDNA and data is expressed as mean±SE.

In the *RAD50* gene we screened four coding regions and exon-intron boundaries among leukemia patients and 504 controls. Two missense variants (p.V315L and p.V127I) were detected in three out of 504 controls. The p.V127I in exon 4, detected twice, and the p.V315L in exon 7 in the *RAD50* gene, detected once, were predicted to be tolerated using the Variant Effect Predictor (SIFT, PolyPhen) tools [23]. In addition we were able to detect c.551+19G>A (rs17166050) single nucleotide polymorphism in the intronic sequence of exon 4 in the *RAD50* gene. The percentage of leukemia cells in the samples obtained from children with acute leukemia had no impact on the detection of polymorphism. The frequency of the A allele of *RAD50*_rs17166050 was significantly different in the controls compared to the leukemia group (ALL+AML) (*p*<0.0019) (Table 1). The frequency of the AA and the GA genotypes was higher in controls compared to patients with ALL (*p<*0.0023, OR=0.3596 (95% CI: 0.1825-0.7083) and *p*<0.0552, OR=0.7051 (95% CI: 0.4929-1.009) respectively). The *RAD50*_rs17166050 variant allele was linked to decreased ALL risk (*p*<0.0009, OR=0.6358 (95% CI: 0.4854-0.8327). Given the result and the close localization of the rs17166050 to the donor site, we performed a molecular test to examine whether this substitution was causing aberrant splicing of *RAD50* pre-mRNA. For that purpose, we isolated RNA from four carriers of the GG genotype, four carriers of the GA genotype and two carriers of the AA genotype. To analyze the length of the *RAD50* cDNA we performed RT-PCR. The forward and reverse primers were complementary to exons 3 and 6 to confirm that amplification product was synthesized from mRNA template and not from genomic DNA. One PCR product of the 602bp length corresponding to correctly spliced exons 4 and 5 was synthesized in each cDNA sample (Figure 1). Differential allelic expression (DAE) showed that the variant allele of *RAD50*_rs17166050 is expressed at a significantly lower level than the wild-type allele. All cDNA samples from heterozygous carriers of the c.551+19G>A variant, except for one individual (no.23), showed an excess of the reference allele over the variant allele (Figure 2). The significant deviation from 1:1 ratio was observed (Mean ± SD, 1.32 ± 0.162534). The Wilcoxon signed-ranks test proved the statistically significance of the results (*p*<0.0001). In our leukemia groups, the previously known sequence variants in the 3rd, 4th, 5th and 7th exon of the *RAD50* were not observed.

**Table 1:** **The allele frequency distribution and results of logistic regression analysis (odds ratio OR and 95% confidence interval CI) of the studied *RAD50 *c.551+19G>A gene variant in controls and acute leukemia patients**

			**Leukemia n=220**		**Controls n=504**	**OR [95 % CI]**		***p***	
**Gene**	**Variant**	**Allele**	**ALL**	**AML**		**ALL**	**AML**	**ALL**	**AML**
***RAD50***	**c.551+19G>A**	G	285	44	671	1^a^	1^a^		
		A	91	20	337	0.6358 [0.4854-0.8327]	0.9104 [0.5250-1.560]	0.0009^1^	0.7194
			**ALL+AML**					**ALL+AML**	
***RAD50***	**c.551+19G>A**	G	329		671	1^a^			
		A	111		337	0.6718 [0.5222-0.8642]		0.0019^1^	

^a^Reference category.

^1^Result statistically significant (*P*<0.05), OR (95% CI) -Odds ratio (95% confidence interval).

The screening of the exon 5, 9, 10, 14, and 19 and the flanking regions in *MRE11* gene in DNA samples from 220 leukemia patients and from blood spots did not reveal any previously described or newly detected molecular variants.

In this study, exons 2, 3, 5, 6, 10 and 13 of the *NBN* gene, in which most of the already known molecular variants occur, were analyzed in 78 leukemia cases (ALL=46, AML=32). We confirmed the occurrence of p.I171V mutation in the 5th exon of the *NBN* gene in two out of 32 AML cases. Because of the high frequency of the p.I171V mutation, the control group was extended to 2400 individuals. Taking together, the results from the current molecular analysis of the *NBN* gene and the previously described mutations and polymorphisms, we still observe a higher incidence of the p.I171V mutation in acute lymphoblastic leukemia (5/188) than among controls (12/2400). The results were statistically significant (Table 2). The four previously described polymorphisms of the *NBN* gene: c.102G>A, c.553G>C, c.1197T>C and c.2016A>G were detected. The distributions of genotype frequencies for each of the analyzed polymorphisms among studied groups are shown in Additional file 2: Table S2. Among these the CC genotype of the c.553G>C (rs1805794) polymorphism was more frequent in leukemia samples, but the results were not statistically significant (*p*=0.0534).

**Table 2:** **Statistical analysis of the occurrence of *NBN *p.I171V mutation in leukemia patients and controls**

		**Leukemia n=220**		**Controls n=2400**	**OR [95% CI]**		***p***	
**Gene**	**Mutation**	**ALL**	**AML**		**ALL**	**AML**	**ALL**	**AML**
***NBN***	**p.I171V**	5	2	12	5.437 (1.894 – 15.60)	21.60 (4.631 – 100.7)	0.0004^1^	0.0001^1^
		**ALL+AML**			**ALL+AML**		**ALL+AML**	
***NBN***	**p.I171V**	7		12	6.540 (2.547 – 16.79)		0.0001^1^	

^1^Result statistically significant (*P*<0.05), OR (95% CI) -Odds ratio (95% confidence interval).

## Discussion

Our previous studies have provided evidence that the p.I171V mutation in the *NBN* gene is a risk allele in acute lymphoblastic leukemia in children and in breast, colorectal, and larynx cancer in adult [6-9]. Some specific haplotypes of the *NBN* gene may be associated with childhood leukemia [13]. Therefore it was interesting to question whether also *MRE11* and *RAD50* germline mutations may increase the risk for childhood leukemia. So far, no studies have addressed this subject. In the current study we screened the selected regions, of the *MRE11*, *RAD50* and *NBN* gene, where most of already known molecular variants occur, among 220 childhood leukemia samples and controls.

In the *MRE11* gene we examined the frequency of the molecular variants in 6 exons and in the exon/intron boundaries. Our study indicated that p.S104C, p.R503H, p.R305W, fs.690–691 or p.R572Q were absent in our cohort of leukemia patients or control DNA. Unfortunately, no data concerning the *MRE11* mutations incidence in childhood leukemia have been reported to date. Our results contradict those obtained with adult cancers. Aberrant reduction or loss of the MRN complex due to an *MRE11* mutation has been associated with several types of cancer including breast carcinoma, ovarian, colorectal, gastric and prostate, leukemia and melanoma [3,17-19,26-28]. For *MRE11* alternations there is too little data to reliably assess their role as cancer risk variants. Importantly several point mutations occur on Nbs1 binding site of the hMRE11 protein, which may highlight the significance of the MRN assembly for DNA damage signaling and repair [29].

In the screened fragments of the *RAD50* gene we detected two molecular variants in three out of 504 controls (2/504 p.V127I and 1/504 p.V315L). These missense variants have been reported previously in the UK familial breast cancer patients (one p.V127I, two p.V315L carriers among 435 cases) but not in controls [15]. In our former study, the p.V127I variant was observed in one out of 280 breast cancer patients and in one individual among 328 controls [16]. The *in silico* analysis did not reveal possible damaging functional effect of these two variants on the RAD50 protein. The rare occurrence and functional evaluation of these variants suggest that none of them is a childhood leukemia risk allele.

In the intronic sequence of the exon 4 we genotyped the c.551+19G>A single nucleotide polymorphism. The distributions of allele frequencies for the analyzed polymorphism among studied groups are shown in Table 1. The *RAD50*_rs17166050 variant allele was linked to decreased ALL risk (*p*<0.0009, OR=0.6358 (95% CI: 0.4854-0.8327). In our previous study [16] concerning the distribution of the c.551+19G>A in breast cancer patients the frequency of the variant and reference allele was similar in both groups. An association of the G allele of the *RAD50* SNP with Crohn's disease (CD) susceptibility has also been reported [30]. However, the authors pointed out that the association of the *RAD50*_rs17166050G with CD appears to be dependent on the presence of the IGR2063 risk allele and may therefore be a result of long-range LD in the study. According to the Reference SNP (refSNP) Cluster Report for rs17166050, MAF (Minor Allele Count) of the A has a frequency of 16.8% in the 1000Genome phase. Thus, individuals with the variant allele may benefit from a protective effect against cancerogenesis. The results of the *in silico* analysis showed that this is the intron variant of the *RAD50* gene with the transcript feature type (ENCODE, VEP). To show whether the variant allele has any function we carried out two molecular tests on cDNA from leukemia patients (n=28) and from healthy individuals (n=12). The first test based on the analysis of the cDNA length in carriers of the AA, GA, GG genotypes to check whether the variant allele could cause aberrant splicing of the *RAD50* pre-mRNA did not reveal any abnormality of *RAD50* cDNA. In these individuals we only observed a 602bp product, which reflected normal splicing (Figure 1). Thus, the heterozygous GA and homozygous AA genotype of the rs17166050 variant do not affect splicing of exon 4 and 5. With respect to intronic variant occurring in human genome we carried out the allele specific real time PCR. The results of the experiment showed different levels of the expression between the reference G and variant A allele. The results were statistically significant. cDNA samples showed an excess of G allele over the A allele consequently (Figure 2). The significantly altered, in a gene dose-dependent manner, mRNA expression level for the *RAD50* gene has been reported in a study dedicated to copy number alternations in adult AML genomes [31]. In childhood acute leukemia we cannot exclude the copy number variations of the *RAD50* gene as mechanism for increasing risk. The interpretation of the current results and explanation of the mechanism underlying the overexpression of the G allele among cancer patients is limited by the rather low number of data and requires further evaluation. The screening of the sequence variations in the 3rd, 4th, 5th and 7th exon of the *RAD50* gene pointed out that p.I94L, IVS3-1G>A, c.687delT, p.R224H and p.Q350X were not observed in our leukemia patients or controls*.* Many reports described the contribution of the *RAD50* gene variants to breast cancer susceptibility in various populations. In the spectrum of investigated mutations of the *RAD50* gene the c.687delT is the most interesting because of contradictory data. The mutation generates a truncated protein without the C-terminal site and has been recognized as a risk factor of familial and sporadic breast cancer in Finnish population [14]. The occurrence of the *RAD50* 687delT mutation in familial/non familial breast cancer in Polish, UK, French and Chinese populations has not been confirmed [15,16,32,33]. Similar frequency of this mutation in cancer patients and controls and the lack of segregation with cancer suggest that it does not increase the risk of cancer development.

Considering together our results from the current and previous studies [6,13] of the *NBN* gene mutations and polymorphisms, we still observed the higher incidence of the p.I171V mutation in acute lymphoblastic leukemia (5/188) than among controls (12/2400) (p<0.0004). In this report we detected the p.I171V mutation, in the 5th exon of the *NBN* gene, in two out of 32 AML cases (p<0.0001). In a recent article, Ciara et al. [34] indicated that heterozygous carriers of the p.I171V and c.657del5 germline mutations in *NBN* gene may demonstrate increased risk of childhood medulloblastoma. In our previous study on the role of the p.I171V mutation as a cancer risk factor for malignant solid tumors in children we obtained contrary results [35], but owing to low number of patients possible Type II error may have occurred. On the other hand our investigation of the cells from heterozygous carriers of the p.I171V, c.657del5 and p.R215W mutation has showed that alone, they do not play an essential role in carcinogenesis [36]. Among the *NBN* polymorphisms, the non-synonymous c.553G>C polymorphism (E185Q) has been the most studied, but results have been inconsistent. In this study we observed that variant allele of the c.553G>C *NBN* polymorphism is more frequent in leukemia cases with no statistical significance. In our previous study we have not observed that association, probably owing to lower number of studied patients. The number of patients can determine the results if we are looking for any association of the low-to-moderate cancer susceptibility gene. The c.553G>C polymorphism has been previously evaluated in relation to acute lymphoblastic leukemia in a Chinese population [37]. In another study the meta-analysis has suggested that the *NBN* c.553G>C variant genotypes might be associated with an increased risk of cancer, especially in Caucasians [12]. Yao and colleagues have excluded any association of the abovementioned polymorphisms and breast cancer risk in any of the populations analyzed [38]. The discrepancy between reported results could be explained by ethnic differences and a possible contribution of other variants of the gene in different populations.

## Conclusion

In conclusion, although we observed that the *RAD50*_rs17166050 variant allele is linked to decreased ALL risk (*p*<0.0009, OR=0.6358 (95%CI: 0.4854-0.8327)), no association of the previously detected mutations in the *RAD50* and *MRE11* genes has been found. It is possible, however, that heterozygous mutations may increase cancer risk in cooperation with other factors including mutations in other genes involved in DNA repair. The discrepancy between our results in childhood leukemia and other studies in adult cancer could be explained by different gene-gene interaction and needs further investigation.

## Abbreviations

MRN: MRE11/RAD50/NBN complex; ALL: Acute lymphoblastic leukemia; AML: Acute myelogenous leukemia; PCR-MSSCP: PCR multi-temperature single-strand conformation polymorphism.

## Competing interests

The authors declare that they have no competing interests.

## Authors’ contributions

MM conceived the study, made substantial contributions to the conception and design of the study, and drafted the manuscript. IZS participated in the design of the study, performed the screening of the *MRE11* gene and sequence analysis and has been substantially involved in statistical analysis. KN participated in screening of the genes and was involved in interpretation of the results. ADK participated in the design of the allele specific real time PCR and interpretation of the results. DJL has collected and selected the patients group. JN participated in the design of the study and has been involved in drafting and critically revising the manuscript. All authors have read and approved the final version of the manuscript.

## Pre-publication history

The pre-publication history for this paper can be accessed here:

http://www.biomedcentral.com/1471-2407/13/457/prepub

## Supplementary Material

Additional file 1 Sequences of the primers. Click here for file

Additional file 2 **The genotype frequency distribution and results of logistic regression analysis (odds ratio OR and 95% confidence interval CI) of the studied *NBN *gene polymorphism in controls and leukemia patients.** Click here for file
