# Supplementary material for: Germline variants in MRE11/RAD50/NBN complex genes in childhood leukemia
Source: BMC Cancer. 2013 Oct 5;13:457. doi: 10.1186/1471-2407-13-457 (PMC3851537; doi:10.1186/1471-2407-13-457)
Supplement: Additional file 1 — Sequences of the primers. [file 1471-2407-13-457-S1.pdf]

Additional file 1: Table S1 Sequences of the primers

***MRE11* gene:**

Exon	Forward 5'→3'	Reverse 5'→3'	Length bp
5	TGCAGTTTGCCTATGATTGC	AAGGCATGCTTTCCACAGAC	262
9	GAA AGC TTT CGT TTG CAC A	CCT TAC AGG CTT CAT GAG	369
10	AAGCTGCTATTCAGCCAAGTT	CCGATGGTGATTGCTCTTCT	275
14	ATT GTA GCC CCT TGT TTT A	AGA CCT ATG GAC TGA CTA TT	231
15	CCACTGTGTCAGCCTCCTTT	TTCCACTCAACTGCCAAGTG	395
19	GAA AAA CTT AGC CAG AAA G	CAG CAA CTA GCT GGC AGT	231

***RAD50* gene**:

Exon	Forward 5'→3'	Reverse 5'→3'	Lenght bp
3	TGCCTTTTTCTCAGAACCAAC	GAAAACAACCATCAACTTACAGACC	296 pz
4	TAAGCAATAGAATAGATACACTG	TTCAATAACTTAATGGACTACAA	295 pz
5	GTGACAGCATAATATCCCACTG	TTGATTTAGCCAGTCCACGA	387 pz
7	GGTTTGGTTTATATTTGATACCTCA	TCCTTGGAGATTCTTCATTCA	324 pz

***NBN* gene**:

Exon	Forward 5'→3'	Reverse 5'→3'	Lenght bp
2	TGTGTATGTGTCTATCAACTTATC	CAA CCC CCT TAC TGGAAACTA	228 pz
5	TTATGGATGTAAACAGCCTC	TACCGAACTATAACACAGCA	328pz
6	CAGATAGTCACTCCGTTTACAA	ACAACTACTGATAAGAGTTA	273pz
7	CCAAATCAAATTCTTATGTGTTCAA	CCGGAGCCAAAAAGAAATTA	168pz
7	CGGGAACGTGTGTTGTTGAT	GAGCAAGACTCCGTCTCAAAA	231pz
10	TTCTCTATTAAAGTTGCTGTA	GATAGTTTGGGATTCTCAT	235pz
10	AGGAGTCCTGCAAAACAAGC	GACAATACCATTCTACAACAG	284pz
13	AGATTCCCAAATGACAAGTG	TTAGCATCACTGGTATCTC	299pz
