# Supplementary material for: Germline variants in MRE11/RAD50/NBN complex genes in childhood leukemia
Source: BMC Cancer. 2013 Oct 5;13:457. doi: 10.1186/1471-2407-13-457 (PMC3851537; doi:10.1186/1471-2407-13-457)
Supplement: Additional file 2 — The genotype frequency distribution and results of logistic regression analysis (odds ratio OR and 95% confidence interval CI) of the studied NBN gene polymorphism in controls and leukemia patients. [file 1471-2407-13-457-S2.pdf]

Additional File 2.

The genotype frequency distribution and results of logistic regression analysis (odds ratio OR and 95% confidence interval CI) of the studied *NBN* gene polymorphism in controls and leukemia patients.

**Gene**	**Polymorphism**	**Genotype**	**Leukemia *n* [%]**	**Controls *n* [%]**	**OR [95% CI]**	***P***
NBN	**c.102G>A**	GG	90 [41]	116 [42]	1[a]	
		GA	95 [43]	129 [47]	0.9492 [0.6477-1.391]	0.7891
		AA	35 [16]	30 [11]	1.504 [0.8588-2.633]	0.1521
	**c.553G>C**	GG	96 [41]	111 [40]	1[a]	
		GC	92 [39]	134 [49]	0.7938 [0.5424-1.162]	0.2345
		CC	44 [20]	30 [11]	1.696 [0.9896-2.906]	0.0534
	**c.1197T>C**	TT	71 [34]	96 [35]	1[a]	
		TC	96 [46]	129 [47]	1.006 [0.6712-1.509]	0.9760
		CC	40 [20]	50 [18]	1.082 [0.6451-1.814]	0.7658
	**c.2016A>G**	AA	92 [43]	115 [42]	1[a]	
		AG	89 [41]	128 [46]	0.8691[0.5913-1.278]	0.4753
		GG	35 [16]	32 [12]	1.367 [0.7869-2.375]	0.2661
